# Geographical predisposition influences on the distribution and tissue characterisation of eccentric coronary plaques in non-branching coronary arteries: cross-sectional study of coronary plaques analysed by intravascular ultrasound

**DOI:** 10.1186/s12947-016-0090-3

**Published:** 2016-11-22

**Authors:** Hidenori Komiyama, Hitoshi Takano, Shunichi Nakamura, Masamichi Takano, Noritake Hata, Miyauchi Yasushi, Yoshihiko Seino, Kyoichi Mizuno, Wataru Shimizu

**Affiliations:** 1 Cardiovascular Medicine, Nippon Medical School, Tokyo, 113-8603 Japan; 2 Cardiovascular Centre, Nippon Medical School Chiba Hokusou Hospital, Tokyo, 270-1613 Chiba Japan

**Keywords:** Atherosclerosis, Plaque distribution, Virtual histology, Vulnerable plaque

## Abstract

**Background:**

We investigated the influence of geographical predisposition on the spatial distribution and composition of coronary plaques.

**Methods:**

Thirty coronary arteries were evaluated. A total of 1441 cross-sections were collected from intravascular ultrasound (IVUS) and radio-frequency signal-based virtual histology (VH-IVUS) imaging. To exclude complex geographical effects of side branches and to localise the plaque distribution, we analysed only eccentric plaques in non-branching regions. The spatial distribution of eccentric plaques in the coronary artery was classified into myocardial, lateral, and epicardial regions. The composition of eccentric plaques was analysed using VH-IVUS.

**Results:**

The plaque was concentric in 723 sections (50.2%) and eccentric in 718 (49.9%). Eccentric plaques were more frequently distributed towards the myocardial side than towards the epicardial side (46.7 ± 7.5% vs. 12.5 ± 4.2%, *p* = 0.003). No significant difference was observed between the myocardial and lateral sides (46.7 ± 7.5% vs. 20.8 ± 5.0%) or between the lateral and epicardial sides. Eccentric thin-capped fibroatheromas were more frequently distributed towards the myocardial side than towards the lateral side (*p* = 0.024) or epicardial side (*p* = 0.005).

**Conclusion:**

Geographical predisposition is associated with distribution, tissue characterisation, and vulnerability of plaques in non-branching coronary arteries.

## Background

The central mechanism of atherosclerosis is chronic inflammation in the presence of damaged vascular endothelium and lipid-laden foamy macrophages derived from infiltration of monocytes into the arterial wall. This mechanism can lead to coronary stenosis and thrombotic obstruction after disruption of the resulting atherosclerotic plaque [1]. Accumulation of leukocytes and lipids, and proliferation of smooth muscle cells, cell death, and fibrosis occur on the damaged endothelium [2]. Although the arterial wall is exposed to risk factors, such as systemic hypertension, hypercholesterolaemia, and diabetes, atherosclerotic plaques develop preferentially at specific areas [3]. In patients with acute coronary syndrome (ACS), the distribution of ruptured coronary artery plaques in the lumen is significantly more eccentric than that of non-ruptured plaques. This finding suggests that blood flow influences the location of ruptured plaques and may even contribute to plaque rupture [4]. The relationship between the spatial distribution and the phenotype of plaques under conditions where blood flow influences atherosclerosis in stable patients has not been fully elucidated. In this study, we used grey-scale intravascular ultrasound (IVUS) to identify spatial plaque distribution, and virtual histology (VH)-IVUS to evaluate the plaque phenotype in 30 consecutive patients who underwent elective percutaneous coronary intervention (PCI), in an attempt to clarify the association between geographical predisposition and plaque phenotype.

## Methods

### Study population

This cross-sectional observational study was carried out in a single centre. We studied 30 consecutive patients who underwent elective PCI under the diagnosis of stable effort angina pectoris and from whom satisfactory grey-scale and VH-IVUS images were obtained. This study was approved by the Nippon Medical School institutional review board, and informed consent was obtained from all patients.

### IVUS image acquisition and analysis

According to our standard protocol and previous report [5], all patients without contraindications were administered aspirin (100 mg/day) and ticlopidine (100 mg B.I.D.) for at least 7 days before the procedure. Per the protocol, clopidogrel (75 mg/day) was also administered in some cases, for at least 4 days before the procedure. At the start of the procedure, weight-adjusted intravenous heparin was given with a target activated clotting time of >250 s. All patients underwent IVUS imaging before any catheter-based intervention, and none of the patients had undergone prior intracoronary intervention in the target vessel. All the lesions were located in native coronary arteries, not in grafted vessels. Intracoronary nitroglycerin (100–200 mg) was administered during all IVUS studies before imaging.

Grey-scale and VH-IVUS images were acquired using a phased array 20 MHz, 3.2 Fr IVUS catheter (EagleEye; Volcano Corporation, Rancho Cordova, CA, USA) with an automated pullback of 0.5 mm/s. The IVUS catheter was tracked over a 0.014-inch guide wire up to a position distal to the diseased segment. The VH-IVUS data were recorded onto the imaging system’s hard disk, and analyses were performed independently by experienced analysts. The analysts were unaware of the angiographic findings and the patients’ baseline clinical and lesion characteristics. All measurements were derived automatically using Volcano imaging system pcVH 2.1 software. The VH-IVUS data analysis was based on grey-scale border contour calculation, and the tissue maps were provided by the software (green = fibrous, yellow = fibro-fatty, red = necrotic core, and white = dense calcium). All cross-sections located near a side branch (within twice the vessel diameter) were excluded from analysis to minimise confounding by flow turbulence. The plaque eccentricity index was the ratio of maximum to minimum plaque thicknesses calculated as previously suggested [6]. An eccentric lesion was defined by an eccentricity index of ≥3, or by the presence of an arc of disease-free arterial wall within the lesion. A three-layered appearance with an intimal thickening of <0.2 mm was considered the upper limit of a ‘normal’ arterial wall [7]. Cross-sections with excessive calcification (calcium arc ≥90°) were excluded from the analysis because of acoustic shadowing of deeper structures, precluding accurate analysis of the vessel area. Lesions with <90° of calcium arc were analysed by extrapolation, assuming that the vessel circumference was circular, and by axial movement of the transducer to identify the vessel area of adjacent non-calcified segments, as described previously [8].

**Fig. 1:**
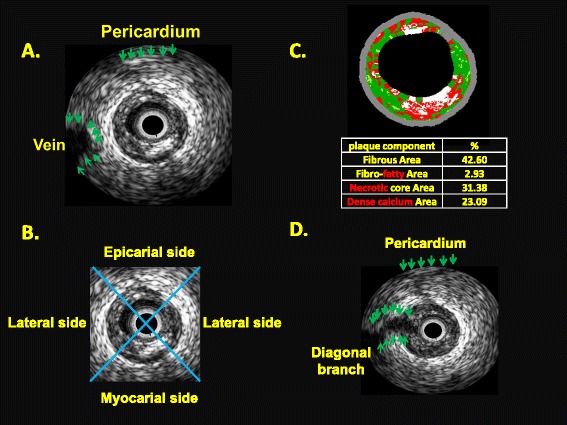
Intravascular ultrasound (IVUS) images of plaque. **a** Landmarks for IVUS orientation. The pericardium appears as a bright and relatively thick structure with varying degrees of spokelike reverberations created by the interwoven fibrous strands. The cardiac vein was observed on the left side of left anterior descending artery. **b** Orientation of plaque distribution was determined by IVUS landmarks. The eccentricity index of the plaque was 3, and it was classified as myocardial plaque. **c** The virtual histology (VH) analysis of plaque composition was divided into four elements (fibrous area, fibro-fatty area, necrotic core, and calcified area). **d** This cross-section was excluded from analysis because of diagonal branching

Perivascular IVUS landmarks—coronary veins, pericardium, myocardium, and side branches—were used for vessel orientation, as previously described [9]. Based on these landmarks, the vessel was divided into myocardial (inner curve of the vessel), epicardial (outer curve of the vessel), and two lateral (intermediate) quadrants. All cross-sections with eccentric plaque distribution were classified according to whether their plaque orientation was centred on the pericardial, myocardial, or either lateral side of the vessel (Fig. 1). In cases where the plaque angle exceeded 90° or the plaque was distributed in between two quadrants, the quadrant with the greater plaque thickness was selected for grouping.

VH-thin-cap fibroatheroma (VH-TCFA) was defined according to a previous study, in which VH-TCFA was a plaque burden (plaque area/external elastic membrane area) exceeding 40% over three consecutive frames, with a confluent necrotic core whose arc was in contact with the lumen for 36° along the lumen circumference [10]. We counted the cross-sections of VH-TCFA morphology and expressed the total as a percentage of all distributed eccentric plaques in each individual patient.

All IVUS images were analysed by two experienced investigators who were blinded to the angiographic data and clinical presentations. When discordance occurred between the observers, a consensus reading was obtained from another investigator.

### Statistical analysis

All data were analysed using SPSS version 21.0 (IBM, Corp, Armonk, NY). All data were analysed by the Shapiro-Wilk test for distribution of normality, and the test showed that the data were not distributed normally. The data were analysed by a non-parametric statistical method, the Kruskal-Wallis test. The post-hoc multiple comparison was performed automatically in SPSS. Data are presented as mean ± SEM. Statistical significance was considered as *p* < 0.05.

## Results

The baseline characteristics of the patient population are presented in Table 1. Of the 30 vessels subjected to IVUS morphometric analysis, 11 were left anterior descending, 8 were left circumflex, and 11 were right coronary arteries. A total of 1441 cross-sections were analysed, comprising 497 (34.5%) in the left anterior descending artery, 325 (22.6%) in the left circumflex artery, and 619 (43.0%) in the right coronary artery.

**Table 1:** Baseline clinical characteristics of patients

Total patients *n* = 30	Number	Percent
Male	23	76.7
Age, mean ± SEM, years	68.8 ± 6.4	
Effort angina pectoris	26	86.7
Unstable angina	1	3.3
Silent myocardial ischaemia	1	3.3
Old myocardial infarction	1	3.3
Ischaemic cardiac myopathy	1	3.3
Risk factors		
Hypertension	26	86.7
Diabetes mellitus	14	46.7
Hyperlipidaemia	20	66.7
Current smoker	17	56.7
Family history	8	26.7
Obesity	10	33.3
Hyperglycaemia	1	3.3

### Orientation of distributed atherosclerotic plaque

**Fig. 2:**
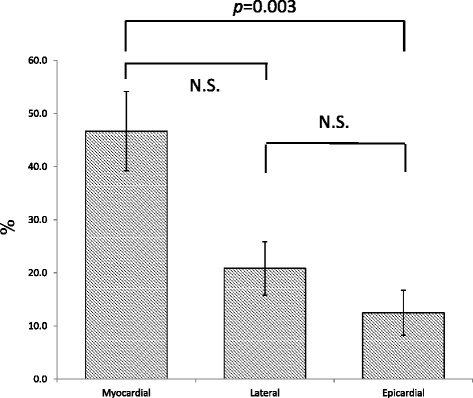
Plaque distribution (%) of the intravascular ultrasound (IVUS) cross-sections in the four quadrants. Of the total plaques, 46.7 ± 7.5% were distributed towards the myocardial side, 20.8 ± 5.0% towards the lateral side, and 12.5 ± 4.2% towards the epicardial side. Data are shown as mean ± SEM. N.S., not significant

The results of the grey-scale IVUS data are summarised in Table 2. Plaque distribution was found to be concentric in 723 (50.2%) and eccentric in 718 (49.8%) cross-sections. Imaging of landmarks, such as the pericardium, one or more accompanying veins, and side branches, allowed for spatial orientation [9]. Of the 718 eccentric plaques, 401 cross-sections were oriented towards the myocardial side, compared with only 80 that were oriented towards the epicardial side, and 237 that were oriented towards the two lateral quadrants. With regard to the plaque distribution in each individual patient, plaque was more frequently oriented towards the myocardial side (50.2 ± 7.0%) than towards the epicardial (25.6 ± 5.4%) or lateral (14.2 ± 4.4%) side (Fig. 2). Eccentric plaques were more frequently distributed towards the myocardial than towards the epicardial side (*p* = 0.003). The minimum vessel diameter was significantly smaller at sites where lateral side plaque was observed, compared with sites with epicardial side plaque. The maximum vessel diameter was significantly smaller at sites with lateral side plaque than at sites with myocardial side plaque. The plaque area of lateral side plaques was significantly smaller than that of myocardial or epicardial plaques.

**Table 2:** Grey-scale IVUS data

	Myocardial side	Lateral side	Epicardial side
Number of cross-sections (number)	401	237	80
Minimum lumen diameter (mm)	2.55 ± 0.03	2.51 ± 0.05	2.59 ± 0.07
Minimum vessel diameter (mm)	4.17 ± 0.04	3.92 ± 0.05*	4.26 ± 0.07*
Maximum lumen diameter (mm)	3.14 ± 0.04*	2.98 ± 0.06*	3.13 ± 0.08
Maximum vessel diameter (mm)	4.66 ± 0.04	4.38 ± 0.06	4.70 ± 0.07
Average lumen diameter (mm)	2.84 ± 0.03	2.73 ± 0.05	2.84 ± 0.07
Average vessel diameter (mm)	4.43 ± 0.04	4.15 ± 0.05	4.48 ± 0.07
Lumen area (mm^2^)	6.72 ± 0.16	6.42 ± 0.25	6.71 ± 0.38

(*p < 0.05)

### Composition of eccentric plaques analysed by VH

We analysed the composition of eccentric plaque by using VH-IVUS and classified it into four categories: Fibrous, fibro-fatty, necrotic core, and calcification. The results are summarised in Table 3. The plaque area of lateral side plaques was significantly smaller than that of myocardial or epicardial plaques. Lateral and epicardial plaques contained significantly more fibrous plaque component than myocardial plaques. Myocardial side plaques contained less fibrous component than lateral and epicardial side plaques, whereas myocardial side plaques contained more fibro-fatty area than lateral plaques. Myocardial and lateral side plaques contained more necrotic core component than the epicardial side plaques, and epicardial plaques contained more calcium than lateral plaques.

**Table 3:** VH-IVUS data

	Myocardial side	Lateral side	Epicardial side
Plaque area (mm^2^)	6.10 ± 0.22*	4.78 ± 0.17*^†^	6.31 ± 0.33^†^
Plaque area (%)	44.93 ± 1.08	43.72 ± 1.18	47.66 ± 2.06
Fibrous area (%)	55.08 ± 0.76*^†^	60.93 ± 0.94^†^	60.48 ± 1.71*
Fibro-fatty area (%)	14.25 ± 0.51^†^	9.83 ± 0.41^†^	11.22 ± 0.69
Necrotic core area (%)	18.26 ± 0.45^†^	18.02 ± 0.61*	15.33 ± 0.97*^†^
Calcified area (%)	11.91 ± 0.72	9.96 ± 0.65*	12.98 ± 1.23*

(*p < 0.05, ^†^ *p* < 0.01)

### Distribution of VH-IVUS-defined TCFAs

**Fig. 3:**
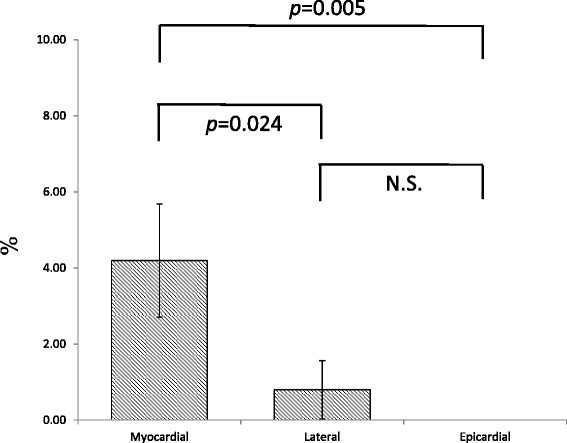
Distribution of thin-capped fibroatheromas (TCFAs). Of the total TCFAs, 4.19 ± 1.49% were distributed towards the myocardial side, 0.80 ± 0.77% towards the lateral side, and none towards the epicardial side. Data are shown as mean ± SEM. N.S., not significant

We observed TCFAs significantly more frequently in myocardial side plaques (4.99 ± 1.61%) than in lateral side plaques (0.80 ± 0.77%, *p* = 0.024) or in epicardial side plaques (0%, *p* = 0.005) (Fig. 3).

## Discussion

The major findings of this study are as follows. (1) Eccentric plaques were more frequently distributed towards the myocardial side than towards the lateral and epicardial sides of the coronary artery. (2) A significant difference was observed in the diameters of the eccentric plaque vessels between the distributed sides. (3) The difference in the plaque component between the distributed sides was also significant. TCFAs were more frequently observed in myocardial side plaques than in lateral or epicardial side plaques.

Coronary arteries are continually subjected to mechanical force, such as tensile or compressive stress and shear stress generated by the heartbeat and pulsatile blood flow during each cardiac cycle [11, 12]. Among the biomechanical forces, flow generates tangential drag force and resultant shear stress. The magnitude of shear stress is determined by changes in luminal geometry, blood flow velocity, and plasma viscosity [13]. Blood flow is disturbed by vessel curvature; it is fast in the outer curvature and slow in the inner curvature. Shear stress is high in the outer curvature and low in the inner curvature [13–15]. Endothelial cells sense shear stress and alter their shape and phenotype [16]. The shear stress is controlled by adapting vessel size to suit the blood flow in response to sustained changes [17]. Hypothetically, if coronary arteries were classified geometrically, myocardial, epicardial, and lateral sides would be exposed to low, high, and intermediate shear stress, respectively. Although shear stress may change over time as plaque progression into the lumen changes coronary flow, we found that eccentric plaques were more frequently distributed towards the myocardial side than towards the epicardial side or lateral side, which is consistent with the hypothesis mentioned above.

Vascular adaptation by shear stress allows the arterial tree to deviate from a straight-tube geometry to another morphology. This phenomenon permits the shear stress to remain unchanged, which provides the predilection site for eccentric plaque development [18]. Human autopsy data showed compensatory enlargement of human coronary arteries in relation to plaque area, and lumen stenosis was delayed until the lesion occupied 40% of the internal elastic lamina, which is termed Glagov’s phenomenon [19]. In the present study, the plaque area in the myocardial and epicardial sides was significantly larger than that in the lateral side, although no significant difference was found in the lumen area between the distributed sides; this finding indicates compensatory enlargement of the myocardial and epicardial vessels. We also confirmed that the minimum vessel diameter was significantly larger on the epicardial side than on the lateral side, and that the maximum lumen diameter was significantly larger on the myocardial side than on the lateral side. Although the difference was not statistically significant, the average vessel diameter was numerically larger on the myocardial side than on the lateral side (4.43 ± 0.04 mm vs. 4.15 ± 0.05 mm, *p* = 0.108), and numerically larger on the epicardial side than on the lateral side (4.48 ± 0.07 mm vs. 4.15 ± 0.05 mm, *p* = 0.0814). The average plaque area was approximately 40% (44.83 ± 0.75%), and the compensatory vascular remodelling was associated with geographical predisposition.

Using VH-IVUS imaging, a spatial relationship between low shear stress and the necrotic core was observed in early plaques (plaque burden <40%) [20], while increases in the necrotic core percentage occurring at the site were typically affected by low shear stress [21]. In serial observations of endothelial shear stress and plaque composition, low-stress segments had greater plaque and necrotic core progression compared with intermediate-stress coronary segments, and high-stress segments had greater necrotic core and calcium progression [22]. In the present study, analysis of plaque composition by VH-IVUS revealed that lateral and epicardial plaques contained significantly more fibrous plaque component than myocardial plaques. Myocardial side plaques contained less fibrous component than the lateral and epicardial side plaques, whereas myocardial side plaques contained more fibro-fatty area than lateral plaques. Myocardial and lateral side plaques contained more necrotic core component than the epicardial side plaques, and epicardial plaques contained more calcium than the lateral plaque. The actual proportion of each plaque component correlated well with assumed shear stress being high on the epicardial side, intermediate on the lateral side, and low on myocardial side (Table 3).

In a previous study using integrated backscatter IVUS [23], Sato et al. reported that in plaques with moderate stenosis in non-branching lesions, lipid pools clustered in the inner curvature and fibrous tissue clustered in the outer curvature. In accordance with their findings, we also found that fibro-fatty and necrotic contents identified by VH-IVUS were more often seen in myocardial side plaque. Although they studied both eccentric and concentric plaques, whereas we selected only the eccentric plaques for analysis, different imaging modalities specifically useful for plaque content characterisation confirmed similar results.

Longitudinal studies in porcine models have shown that TCFAs, which develop more frequently in the coronary regions, are exposed to low shear stress throughout their evolution [24, 25]. Autopsy studies have shown that atherosclerotic lesions are provoked by TCFA rupture, which can lead to thrombosis, ACS, and sudden cardiac death [26, 27]. In vitro studies using the finite element method have demonstrated that the shear stress of the vascular lumen is an important determinant of coronary plaque vulnerability and plaque rupture [28, 29]. Fukumoto et al. demonstrated that localised elevation of blood pressure and shear stress are associated with coronary plaque rupture in the proximal or top portion of the plaque in ACS patients [30]. The shear stress concentration is frequently correlated with the plaque rupture site. Plaque rupture may heal without any symptoms or lead to mural thrombosis with subsequent asymptomatic healing [31, 32].

Although the precise mechanisms that promote the focal formation of rupture-prone coronary plaques in vivo remain to be elucidated, we found that eccentric TCFAs were clustered towards the myocardial side. We only analysed eccentric plaques, which may be predisposing to future coronary events [4]. The relationship between rupture-prone TCFAs and subsequent thrombus formation or clinical events is still unknown [31], as is whether TCFA-induced plaque ruptures lead to lumen stenosis. Although it is also still unclear whether TCFA clusters towards the myocardial side actually rupture and lead to clinical symptoms or lumen stenosis, the method for the geographical classification of coronary plaques by using IVUS in this study is simple and applicable in clinical settings, and can be utilized to characterise the complex profile of atherosclerotic plaque.

### Study limitations

The limitations of this study are as follows. First, the sample size was small; only 30 coronary arteries in 30 patients were analysed. Second, all the patients were in stable condition, and their plaque phenotype may have been different from that of unstable patients. Third, we included right arteries, in which atherosclerotic change may differ from that in left coronary arteries [33]. Fourth, we did not calculate the absolute value of inter-observer variability in identifying the distribution of plaque, although this does not invalidate the findings because discordance in the image reading was rare. Fifth, this study was designed as an observational study, and the clinical importance of geographical predisposition should be assessed prospectively.

## Conclusions

Eccentric coronary plaques are more often observed on the inner side of the coronary arteries. The geographical predisposition of myocardial distribution in the human coronary artery was associated with a larger lipid burden, a thinner fibrous cap, and a higher prevalence of TCFA. The geographical classification of coronary plaques using IVUS is applicable in clinical settings to elucidate the complex profile of atherosclerotic plaque.

## Abbreviations

ACS: Acute coronary syndrome
IVUS: Intravascular ultrasound
PCI: Percutaneous coronary intervention
TCFA: Thin-capped fibroatheroma
VH: Virtual histology
VH-IVUS: Virtual histology intravascular ultrasound
